# Why alternative teenagers self-harm: exploring the link between non-suicidal self-injury, attempted suicide and adolescent identity

**DOI:** 10.1186/1471-244X-14-137

**Published:** 2014-05-22

**Authors:** Robert Young, Nina Sproeber, Rebecca C Groschwitz, Marthe Preiss, Paul L Plener

**Affiliations:** 1 MRC Social and Public Health Sciences Unit, University of Glasgow, Glasgow G12 8RZ, Scotland; 2 Department of Child and Adolescent Psychiatry and Psychotherapy, University of Ulm, Steinhoevelstr. 5, D- 89075 Ulm, Germany

## Abstract

**Background:**

The term ‘self-harm’ encompasses both attempted suicide and non-suicidal self-injury (NSSI). Specific adolescent subpopulations such as ethnic or sexual minorities, and more controversially, those who identify as ‘Alternative’ (Goth, Emo) have been proposed as being more likely to self-harm, while other groups such as ‘Jocks’ are linked with protective coping behaviours (for example exercise). NSSI has autonomic (it reduces negative emotions) and social (it communicates distress or facilitates group ‘bonding’) functions. This study explores the links between such aspects of self-harm, primarily NSSI, and youth subculture.

**Methods:**

An anonymous survey was carried out of 452 15 year old German school students. Measures included: identification with different youth cultures, i.e. Alternative (Goth, Emo, Punk), Nerd (academic) or Jock (athletic); social background, e.g. socioeconomic status; and experience of victimisation. Self-harm (suicide and NSSI) was assessed using Self-harm Behavior Questionnaire and the Functional Assessment of Self-Mutilation (FASM).

**Results:**

An “Alternative” identity was directly (r ≈ 0.3) and a “Jock” identity inversely (r ≈ -0.1) correlated with self-harm. “Alternative” teenagers self-injured more frequently (NSSI 45.5% vs. 18.8%), repeatedly self-injured, and were 4–8 times more likely to attempt suicide (even after adjusting for social background) than their non-Alternative peers. They were also more likely to self-injure for autonomic, communicative and social reasons than other adolescents.

**Conclusions:**

About half of ‘Alternative’ adolescents’ self-injure, primarily to regulate emotions and communicate distress. However, a minority self-injure to reinforce their group identity, i.e. ‘To feel more a part of a group’.

## Background

Self-harm is the collective term for acts of self-injury or self-poisoning with or without suicidal intention. These behaviours are common during adolescence, but share many risk factors. While interlinked suicidal and non-suicidal forms of self-harm are considered as qualitatively distinct from one another. International expert and systematic reviews estimate around 30% of adolescents have suicidal thoughts [1], approximately 4% attempt suicide [2] and around 18% have engaged in non-suicidal self-injury (NSSI; frequently abbreviated to self-injury) [3]. While these estimates are skewed towards European and North American studies, rates from non-western nations are similar. Certain adolescent subpopulations are at even greater risk of self-harm. For example, gay, bisexual, lesbian or transgendered (GBLT) youth are approximately 2–4 times more likely to self-harm than non-GBLT youth [4], ethnic minorities (primarily women) are similarly at risk [5,6], as are teenagers who identify with youth subcultures such as Goth [7-9] or Emo [10,11]. While minority groups are often exposed to additional risk-factors for self harm such as victimisation or low socioeconomic status [4-6] the elevated self-harm rate is often attributed to ‘minority stress’ as a consequence of a stigmatised identity. The reason(s) why certain youth subcultures are more likely to self-harm, particularly NSSI, are unclear and are the focus of this paper.

Alternative youth culture (or subculture) is a difficult term to define both theoretically and practically, nevertheless the description used by British police when recording crimes against minority subcultures is constructive. Greater Manchester police define Alternative subculture as “…a broad term to define a strong sense of collective identity and a set of group-specific values and tastes. This typically centres on distinctive style, clothing, make up, body art and music preference. Those involved usually stand out to both fellow participants and to those outside the group. Groups typically under the ‘alternative’ umbrella include Goths, Emos, Punks and Metallers…” [12].

Adolescents who identify with Alternative youth cultures with predominately ‘dark, sinister disturbing and morbid’ themes such as Goth [7,9], Emo [10,11,13] or Heavy metal [14-16] are reported to engage in a range of self-harming behaviours, but this conclusion is based on a handful of studies or case reports and the validity of these reports has been contested [17-19]. While studies of older subcultures (e.g. Heavy metal) focus on its association with suicide and suicide ideation, research on contemporary subcultures (e.g. Emo) concentrate on its links with NSSI. Contemporary media focus on the links between self-harm and three often conflated Alternative subcultures, namely Emo, Punk and Goth. Accordingly our research will primarily focus on these groups.

While consistent positive, the effect size of the association between self-harm and Alternative subculture varies dramatically between the few relevant studies. A 2001 study found a modest correlation (*r =* 0.13, boys; 0.23 girls) between adolescents preference for Heavy metal or Gothic music and an index of suicide risk based on both suicide attempts and ideation [16], but this became non-significant after adjusting for sociodemographic factors. A 2006 study of young adults (age 19) reported those who strongly identified as Goth were more likely to attempt suicide (46.7%, Goth; 5.4% non-Goth) or engage in NSSI (46.7%, Goth; 3.1% non-Goth) than those who did not, even after adjusting for social background [9]. Empirical research on the links between Emo subculture and self-harm is weak and based on perceived risk [13,19], case study [10], or social media research [11]. Thus, the overall evidence-base in this area is sparse.

Despite being a key feature of identity development during adolescence, psychiatry largely ignores youth culture’s influence on young people’s psychopathology [20]. Thus a number of key questions concerning the link between self-harm (both suicide and NSSI) and youth culture(s) remain unanswered. These include: is the association robust; do other youth identities show a similar association; are ‘Alternative teenagers’ reasons for NSSI the same as other teenagers’; and can any additional risk attributed to membership of a specific youth subculture be explained by social background or other risk factors such as victimisation? This study seeks to answer these questions using a community sample of German teenagers who participated in a study of NSSI.

### Reasons for NSSI

Several studies have investigated the psychological motivation for NSSI [21,22] and their results used to support a four factor explanatory model comprised of *intrapersonal negative reinforcement* (e.g. reduction of negative emotions such as anxiety or anger); *intrapersonal positive reinforcement* (e.g. to relieve feeling numb or empty); *interpersonal positive reinforcement* (e.g. gaining attention or emotional support from peers); and *interpersonal negative reinforcement* (e.g. reduction in victimisation) [23]. Other researchers propose a simpler two factor interpersonal and intrapersonal model [21]. Irrespective of the method of assessment, NSSI performs a clear social and communicative function among teenagers which is arguably linked to their social identity. For example, a study of Emo teenagers argues that online exchanges about self-injury methods, interpersonal and intrapersonal justifications for self-injury are common, as are vivid descriptions of peer victimisation [11].

### Youth identity, psychopathology and self-harm

Both sociology and psychology consider youth culture to be an important phenomenon. Within social psychology youth identity is often termed “adolescent peer crowd affiliation or identification” and is tied to social identity theory [24]. This theory is supported by experiments showing how even minimal identification with a ‘fictional’ social group leads to increased in-group influence and adoption of stereotypical in-group behaviours, particularly among newer members [25]. Western adolescents readily identify with specific ‘social crowds’ and accordingly real social groups should exert considerably greater influence than fictional ones. Common peer crowds include the Brains or Nerds (academic), Jocks (athletic), Burnouts (substance using and antisocial) and Nonconformists (rebelling via unusual clothing or ideas) with each type linked to a specific health behaviour profile. For example, Burnouts and Nonconformists engage in more risky behaviours [26] and Burnouts report poor psychological health [27]. The generic descriptions and associated risk behaviours of the Burnout and Nonconformists overlap with those of Alternative subcultures within the sociological literature.

Within sociology youth subculture is related to classic subculture and deviance theory. These explain youth subculture as a reaction to mainstream (particularly middle-class) values and as a method for marginalised youth to achieve a form of status within a social system that conflicts with their ideals. Contemporary research often focuses on (adult) Punk, Goth and related subcultures and stresses the importance of two areas; out-group distinction and in-group socialisation [28]. *Out-Group Distinction* relates to how in-group values, symbols and practices distinguish members from ‘mainstream’ society, while *In-Group Socialisation* refers to subcultural social ties, conventions, commitment and in-group status. Milner’s qualitative study of high school youth culture confirmed the marginal status of contemporary Alternative teenagers within the (North American) high school ecology, but also describes how membership partially separates and protects them from the wider peer-group hierarchy [29].

If we assume the link between (sub)cultural identity and self-harm is robust then how can we explain the additional risk it confers? Social contagion is often invoked as the explanation for this. This refers to the rapid transmission of behaviours from one person to others via social mechanisms and is usually indicated by the clustering of behaviours within specific social groups [30,31]. It has been reported that 73% of females and 57% of male adolescents who self harm (i.e. with or without suicidal motivation) also have a friend who does so [32] and 82.1% of adolescent inpatients with NSSI reported having a friend who self-injured as well [33]. A recent review of NSSI social contagion confirmed the phenomena in at least 16 studies [34] and identified three mutually compatible mechanisms which may also explain the ‘Alternative-identity effect’. The first proposed mechanism is *Assortive relations* (or selection). Here it is suggested that Alternative teenagers predisposed to self-harm are attracted to subcultures with disturbing and emotional themes that mirror their own experience. The other proposed explanations are *direct imitation*, e.g. Alternative teenagers copy their self-harming friends and *indirect imitation* or *media influence*, e.g. Alternative teenagers mimic subcultural icons’ self-harming behaviours. From a sociological standpoint Alternative teenagers’ nonconformist nature predisposes them to a greater acceptance and understanding of atypical behaviours, which may include one or more forms of self-harm. A further possibility is that the additional risk is a statistical *artefact*, i.e. once we adjust for key risk factors linked to a nonconformist identity such as “victimisation” the alleged effect will lessen or even disappear.

### Aims

This study explores the Alternative-identity effect among contemporary youth and has four specific aims: 1) to replicate past findings linking Alternative teenagers with self-harm (suicide or NSSI) in a contemporary German sample; 2) to determine if other contemporary youth identities are linked with self-harm, either as a risk (Nerd) or protective (Jock) factor; 3) to ascertain if the reasons for NSSI are different to those of Non-Alternative teenagers; 4) to determine if Alternative teenagers’ elevated risk of suicide and NSSI is (partially or wholly) accounted for by known sociodemographic risk factors (e.g. gender, socioeconomic status, migrant) or by victimisation.

## Methods

### Sample details

The participants were 452 German ninth-grade students from 10 schools (30 classes) located in and around the city of Ulm who took part in an anonymous study of attempted suicide and NSSI. Only students who provided both parental (written) and individual (verbal and written) consent participated in the study. Of 748 eligible students, 656 were present at the day of assessment and 452 (68.9%) of the students and their caregivers consented to take part in the study. Information on non-consenting participants is not available as German school regulations forbid the release of student data without consent. The majority of students were aged 14 (n = 112) or 15 (n = 298) although a few were older (age 16, n = 37; age 17, n = 3; missing, n = 2) and 46.2% (n = 209) were female. More than half (n = 249) of students provided information on experience of victimisation in the optional sociometric section. The study was approved by the Institutional Review Board of the University of Ulm and by the local school authorities. Students with self-harm and emotional problems could access help directly from the study team (contact card) and from external sources (local services and information card). However, no students chose to do so.

### Measures

#### Demographics and risk factors

Students provided basic sociodemographic and social background information including: gender; type of school (‘hauptschule’, i.e. vocational; ‘realschule’, i.e. intermediate; ‘gymnasium’, i.e. academic); and migrant background, dichotomised into German (both parents German) or immigrant (1 or 2 parents immigrant) background (Table 1). Socioeconomic status was assigned by asking for parental occupations, calculating a standard household income (based on the data from the German federal statistical bureau [35]) and classifying them into lower, middle and upper-class households [36]. Regarding victimisation, students indicated if in school they ‘were often physically attacked (punched, kicked, pushed)?’ or ‘repeatedly had rumours spread that damaged their reputation’ using a 4-point Likert (true, mostly true, mostly untrue, untrue) scale, with those who gave a ‘true’ or ‘mostly true’ response considered victims of physical or relational bullying.

**Table 1:** Descriptive statistics and sample demographics

**Categorical variables (Base sample = 452)**	**N**	**%**
**Alt (Emo, Goth, Punk) identity (Mv = 7)**		
No identification	412	92.6
Mild^1^	26	5.8
Moderate to complete^1^	7	1.6
**Nerd identity (Mv = 10)**		
No identification	335	75.8
Mild	76	17.2
Moderate to complete	31	7.0
**Jock identity (Mv = 6)**		
No identification	70	15.7
Mild	85	19.1
Moderate to complete	291	65.2
**Sex**		
Female	209	46.2
Male	243	53.8
**Immigrant parent(s)**		
Both parents German	329	72.8
1 or 2 parents immigrant	123	27.2
**School type**		
Vocational	72	15.9
Intermediate	206	45.6
Academic	174	38.5
**Socioeconomic status (Mv = 7)**		
Lower	66	14.8
Middle	281	63.1
Upper	98	22.0
**Physically or relational bullied (Mv = 203)**^2^		
Not victimised	219	88.0
Victimised	30	12.0
**SHBQ Self-injury (Mv = 8)**		
No	352	79.3
Yes	92	20.7
**SHBQ Suicide attempt (Mv = 24)**		
No	410	95.8
Yes	18	4.2
**SHBQ Suicide ideation (Mv = 64)**		
No	289	74.5
Yes	99	25.5
**FASM Self-injury (Mv = 23)**		
No	233	54.3
Yes	196	45.7

^1^Categories collapsed for logistic regression analysis due to small numbers.

^2^Approximately 44% of participants’ did not take part in the sociometric section of the study in which victimisation was assessed.

Mv = Missing values.

### Self-harm (attempted suicide, suicidal ideation and NSSI)

Self-harm was assessed using the Self-Harm Behavior Questionnaire (SHBQ) [37], which is a self-report measure to assess the lifetime prevalence of self-harming behaviours including NSSI (‘Have you ever hurt yourself on purpose? [e.g., scratched yourself with fingernails])’ suicidal ideation (‘Have you ever talked or thought about committing suicide?’), and attempted suicide (‘Have you ever attempted suicide?’), see Table 1. The instrument is validated (Cronbach's α between 0.89-0.96 for 4 subscales) and has been used with both American and German adolescent community samples [3,38]. The German version [39] has also been validated (Cronbach's α between 0.87-0.96). Students also completed the functional Assessment of Self-injury (FASM) instrument which asks about recent (past 12 months) engagement in 12 different forms of NSSI, e.g. ‘cut or carved your skin’. Engaging in any form of NSSI within the last 12 months was classified as recent NSSI. The FASM and SHBQ use a similar method to count the number of acts of self-injury. Students indicated how many acts of self-harm they had engaged in, truncated at ‘4 or more’ acts.

### Reasons for self-injury

The FASM contains 22 items which focus on individual motives for NSSI, all measured on a 4-point frequency (‘almost never’ to ‘almost always’) scale [22]. The instrument possesses reasonable psychometric properties (Cronbach's α 0.65–0.66) [40] and has been used with both English [33,41] and German speaking samples [42,43].

### Youth culture

Students were asked how much they identified with 18 different youth cultures (e.g. Goth, Emo, Hip-hop, etc.) using a 5-point identity scale ('not at all', ‘a little or mildly’, ‘moderately’, ‘strongly’, or ‘I am one’). The scale was translated into German from an existing questionnaire [9,44] and updated to include contemporary subcultures. Students who identified at least ‘mildly’ as Goth, Emo, or Punk were classified as ‘Alternative’. Nerd and Jock ‘peer-crowd’ identity was measured on the same 5-point scale. Due to the frequency distribution Nerd and Jock identities were collapsed into a three categories (none, mild, moderate to complete identification, see Table 1).

### Statistical analysis

Analysis was performed with SPSS 21 using descriptive statistics and both parametric (t-tests) and non-parametric (χ2, Mann–Whitney U exact) inferential statistics. When appropriate, unadjusted p-values and those adjusted for multiple comparisons (Benjamini-Hochberg method) are presented. Correlations were used to assess the strength of association between different identities and different forms of self-harm (attempted suicide, suicidal ideation or NSSI). Differences between Alternative and Non-Alternative youth about motivations for NSSI were determined via t-tests and Mann–Whitney U tests. Principle components analysis with varimax rotation was used to identify factors in the FASM and youth culture questionnaires. Multivariate logistic regression was used to assess the strength of association (odds ratios) between youth identity and different types of self-harm. Estimates from unadjusted models and those adjusted for Jock identity, gender, socioeconomic status, immigrant background and school type were compared. A final model further adjusted for the impact of victimisation. No adjustment for the clustered (school) nature of the data was made due to the small number of schools involved and because estimates of the ICC school effect for all outcomes was extremely low (≤0.02); this is consistent with the literature on school effects on suicide and self-injurious behaviours [45].

## Results

### Sample statistics and demographics

Basic descriptive statistics and demographic characteristics are shown in Table 1. The rates of both attempted suicide (4.2%) and suicidal ideation (25.5%) are typical for an adolescent sample. The rate of NSSI varied according to the instrument; the SHBQ provided a lower ‘lifetime’ rate (20.7%) than the FASM ‘12 month’ prevalence estimate (45.7%). Alternative adolescents were significantly more likely to engage in NSSI (SHBQ, 45.5% vs. 18.8%; χ2 = 13.1, df 1, p < 0.001; FASM, 75.0%; vs. 43.1%, χ2 = 12.1, df 1, p < 0.001), attempt suicide (17.2% vs. 3.3%; χ2 = 12.8, df 1, p < 0.001), or think about attempting suicide (51.9% vs. 23.9%; χ2 = 10.2, df 1, p < 0.001) than Non-Alternative students.

### Association between self-harm outcomes

All self-harm outcomes were significantly correlated, although the magnitude of the association varied considerably (see Additional file 1: Table S2). NSSI, as measured by SHBQ and FASM, were highly correlated (*r* = 0.49), as were their measures of NSSI frequency (*r* = 0.75). The associations between NSSI and attempted suicide were of a moderate to small effect size (SHBQ, *r* = 0.29; FASM, *r* = 0.17), as were the associations between NSSI and suicidal ideation (SHBQ, *r* = 0.29; FASM, *r* = 0.27). The correlation between attempted suicide and suicidal ideation was low (*r* = 0.17), but is consistent with psychometric studies of the SHBQ [37].

### Factor analysis of the youth culture questionnaire

Factor analysis was used to construct continuous measures of subcultural identity. Subcultures with less than 10 members were omitted from further analysis and 12 main music-based subcultures identified. Non-music based subcultures (Nerd and Jock) were largely uncorrelated with the other identities and were excluded. Principle components (varimax) analysis of the 12 music-based identity items produced a clear three factor solution with factors labelled: (1) Alternative; (2) Indie; and (3) Urban identity (Table 2). The reliability of the three putative identity subscales ranged from good to borderline acceptability (Cronbach's α 0.72, Alternative; 0.57, Indie; 0.58, Urban) [46]. By a substantial margin the items with the three highest factor loadings on the Alternative subscale (Emo 0.80; Punk, 0.81; and Gothic, 0.80) were those used to construct our categorical measure of Alternative identity. The two measures were highly correlated (*r* = 0.87).

**Table 2:** **Principle components analysis**^**1**^**of youth subcultural identity**

**Subcultural identity**	**Factor 1**	**Factor 2**	**Factor 3**	**U**	**M**	**SD**
Emo	.81			.67	1.05	0.26
Punks	.81			.71	1.08	0.39
Gothic	.80			.64	1.03	0.22
(Death) Metal	.67			.57	1.24	0.77
(Hard) Rock	.57			.53	1.52	1.01
Hippies		.73		.55	1.19	0.55
Grunge		.67		.47	1.06	0.33
Indie		.65		.45	1.26	0.72
Reggae		.56		.49	1.54	1.06
Techno or Rave			.79	.65	1.51	0.96
Hip Hop			.74	.56	1.92	1.11
Drum & Base			.62	.49	1.41	0.84

^1^Varimax rotation, n = 402, 56.3% of variance explained. Loadings under 0.5 omitted.

Items scored on a 5-point 1–5 identity scale.

### Factor analysis of the FASM

Principle components (varimax) analysis of the FASM with the 170 self-harming students in our study indicated a three factor solution (Table 3). The three factors are similar to those found in a study of self-injuring German psychiatric patients [43] and were labelled: (1) Interpersonal influence and communication, e.g. ‘To receive more attention from your parents or friends’; (2) Automatic functions, e.g. ‘To stop bad feelings’; and (3) Peer avoidance-attraction, e.g. ‘To feel more a part of a group’.

**Table 3:** **Principle components analysis**^**1**^**of reasons for engaging in NSSI**

**Reasons for self-injury**	**Factor 1**	**Factor 2**	**Factor 3**	**U**	**M**	**SD**
8. To receive more attention from your parents or friends	.86			.74	0.18	0.55
17. To get your parents to understand or notice you	.78			.62	0.18	0.62
11. To get other people to act differently or change	.71			.54	0.11	0.42
15. To let others know how desperate you were	.65			.52	0.39	0.84
20. To get help	.64			.42	0.08	0.40
12. To be like someone you respect	.51			.42	0.04	0.19
3. To get attention	.48			.29	0.19	0.54
21. To make others angry	.43			.24	0.08	0.39
14. To stop bad feelings		.72		.55	0.65	0.97
7. To try to get a reaction from someone, even if it’s negative		.71		.54	0.15	0.52
22. To feel relaxed		.70		.52	0.19	0.54
6. To get control of a situation		.69		.51	0.26	0.62
4. To feel something		.69		.55	0.29	0.67
10. To punish yourself		.51		.38	0.33	0.71
2. To relieve feeling numb or empty		.50		.32	0.16	0.51
18. To give yourself something to do when alone				.22	0.18	0.54
13. To avoid punishment or paying the consequences			.73	.54	0.03	0.17
9. To avoid being with people			.56	.43	0.09	0.44
5. To avoid doing something unpleasant you don’t want to do			.56	.46	0.20	0.55
16. To feel more a part of a group	.44		.55	.51	0.05	0.27
1. To avoid school, work, or other activities			.53	.38	0.16	0.53
19. To give yourself something to do with others				.12	0.03	0.17

^1^Varimax rotation, n = 170, 44.6% of variance explained. Loadings under 0.4 omitted. Items scored on a 4-point 0–3 scale.

### Motivation for NSSI among alternative and non-alternative youth

Table 4 reports differences between Alternative and non-Alternative youth in their reasons for NSSI. Almost every type of motivation to self-injury was more frequently endorsed by Alternative than non-Alternative adolescents; the majority (13 of 22) significantly so. In relation to peer group influence notable differences include, ‘To avoid being with people’ and ‘To feel more a part of a group’. Compared to their non-Alternative peers, Alternative-teenagers have significantly higher FASM factor scores for both automatic and interpersonal-communication functions. Unexpectedly we found no difference in relation to the third factor (peer avoidance-attraction) which may indicate a relatively weak or specific effect. P-values adjusted for multiple comparisons were unsurprisingly lower than unadjusted values. Nevertheless, both sets of p-values were substantively no different. Results using non-parametric group comparisons with or without adjustment for multiple testing also gave similar results (Mann–Whitney U, see Additional file 2: Table S1).

**Table 4:** Reason for self-injury by alternative (emo, punk, goth) identification

	**Non-alt identity (n = 145)**		**Alt identity (n = 22)**				**Corrected**
**Reasons for self-injury**	**M**	**SD**	**M**	**SD**	**t**^**1**^	**p-level**	**p-level**^**2**^
8. To receive more attention from your parents or friends	0.13	0.46	0.55	0.91	-3.36	**.001**	**.003**
17. To get your parents to understand or notice you	0.15	0.56	0.36	0.95	-1.49	.138	.192
11. To get other people to act differently or change	0.08	0.31	0.32	0.84	-2.53	**.012**	**.026**
15. To let others know how desperate you were	0.30	0.72	1.00	1.31	-3.72	**≤ .001**	**.002**
20. To get help	0.03	0.22	0.41	0.91	-4.28	**≤ .001**	**≤ .001**
12. To be like someone you respect	0.03	0.18	0.05	0.21	-0.26	.798	.798
3. To get attention	0.14	0.42	0.59	0.96	-3.81	**≤ .001**	**.002**
21. To make others angry	0.06	0.38	0.18	0.50	-1.33	.187	.246
14. To stop bad feelings	0.60	0.95	1.09	1.06	-2.22	**.028**	**.050**
7. To try to get a reaction from someone, even if it’s negative	0.12	0.48	0.36	0.73	-2.01	**.046**	.077
22. To feel relaxed	0.18	0.52	0.32	0.65	-1.12	.263	.313
6. To get control of a situation	0.24	0.60	0.41	0.73	-1.18	.240	.301
4. To feel something	0.21	0.59	0.82	0.96	-4.07	**≤ .001**	**≤ .001**
10. To punish yourself	0.28	0.63	0.73	1.08	-2.81	**.006**	**.013**
2. To relieve feeling numb or empty	0.12	0.45	0.50	0.74	-3.38	**.001**	**.003**
18. To give yourself something to do when alone	0.17	0.54	0.23	0.53	-0.44	.659	.687
13. To avoid punishment or paying the consequences	0.03	0.16	0.05	0.21	-0.46	.649	.687
9. To avoid being with people	0.06	0.33	0.36	0.85	-3.13	**.002**	**.006**
5. To avoid doing something unpleasant you don’t want to do	0.17	0.52	0.41	0.73	-1.88	.062	.097
16. To feel more a part of a group	0.03	0.20	0.23	0.53	-3.27	**.001**	**.004**
1. To avoid school, work, or other activities	0.13	0.48	0.41	0.80	-2.31	**.022**	**.043**
19. To give yourself something to do with others	0.03	0.18	0.00	0.00	0.88	.380	.431
Factor 1: Interpersonal Influence and communication	-0.10	0.72	0.68	1.99	-3.47	**.001**	**.003**
Factor 2: Automatic functions	-0.07	0.92	0.56	1.35	-2.80	**.006**	**.013**
Factor 3: Peer avoidance-attraction	-0.04	0.86	0.31	1.69	-1.55	.124	.183

Note: Significant differences are emboldened.

^1^Equal variances assumed, df = 165. t-test adjusted for nonequal variances give similar, but attenuated results. Items scored on a 4-point 0–3 scale.

^2^Benjamini-Hochberg corrected p-values for multiple testing.

### Association between different youth subcultures and self-harm

Table 5 shows the correlation between subcultural identity factor scores, Alternative, Jock or Nerd identity, self-injury, suicidal thoughts and attempted suicide. Whether measured as a factor score or as the highest level of identification an Alternative identity is consistently associated with lifetime self-injury (SHBQ *r =* 0.20-0.24; FASM *r =* 0.12-0.17), frequency of self-injury (SHBQ *r =* 0.32-0.35; FASM *r =* 0.21-0.29) suicidal thoughts (*r =* 0.13-0.20) and attempted suicide (FASM *r =* 0.25-0.29). Other subcultures were not or only inconsistently and weakly associated with self-harm. Both Indie and Urban identity were weakly correlated with some types of self-harm, e.g. Urban identity and SHBQ self-injury (*r =* 0.15). A Jock identity was inversely associated (*r = -*0.11-0.18) and a Nerd identity uncorrelated with self-injury, attempted suicide and suicidal ideation.

**Table 5:** Correlation between subcultural identity, self-injury, suicidal thoughts or behaviours

**No.**	**Identity or self-harm measures**	**1.**	**2.**	**3.**	**4.**	**5.**	**6.**
1.	Identity factor 1: Alt	-					
2.	Identity factor 2: Indie	.04	-				
3.	Identity factor 3: Urban	.03	-.06	-			
4.	Alt (Emo, Punk, Goth)^1^	.87**	.21**	-.02	-		
5.	Jock or Athlete	-.01	.04	.23**	.01	-	
6.	Nerd	.09	.09	-.09	.07	-.03	-
7.	SHBQ Self-injury	.20**	.12*	.08	.24**	-.11	.03
8.	SHBQ Self-injury frequency	.32**	.13*	.03	.35**	-.17**	.04
9.	FASM Self-injury	.12*	.02	.14*	.17**	-.18**	-.03
10.	FASM Self-injury frequency	.21**	.11*	.04	.29**	-.15*	.00
11.	SHBQ Suicide attempt	.29**	.05	.11	.25**	-.11*	.05
12.	SHBQ Suicide ideation	.13*	.07	.05	.20**	-.12*	.06

Note: leading zeros omitted. * = p-level ≤ .05, ** = p-level ≤ .01. Significant correlations are emboldened. N = 311.

^1^This refers to the highest level of identification with that set of subcultures.

### Association between youth subcultures and self-harm adjusting for covariates

The univariate associations (odds ratios) between self-harm, different youth subcultures and covariates are shown in Table 6 and Table 7. Using categorical measures, Alternative teenagers were far more likely to self-injure (SHBQ OR = 3.6; FASM OR = 3.9), have suicidal thoughts (OR = 3.4), or attempt suicide (OR = 6.0) than their non-Alternatives peers. Adolescents with a strong Jock identity were less likely to think about suicide than non-Jocks (OR = 0.5). As expected, covariates were significantly associated with at least one form of self-harming behaviour or cognition, e.g. females were more likely to self-injure (SHBQ OR = 2.9), as were those with a migrant background (SHBQ OR = 2.1).

**Table 6:** Association between self-injury and alternative identity

	**Self-injury (SHBQ, n = 428)**				**Self-injury (FASM, n = 415)**			
**Predictors**	**OR**	**95% CI**	**Adj OR**	**95% CI**	**OR**	**95% CI**	**Adj OR**	**95% CI**
**Alt-identity**								
None	1.00	-	1.00	-	1.00	-	1.00	-
Moderate or stronger	**3.56**	**1.71-7.38**	**4.16**	**1.90-9.11**	**3.92**	**1.72-8.95**	**4.04**	**1.73-9.44**
**Jock-identity**								
None	1.00	-	1.00	-	1.00	-	1.00	-
Moderate	0.74	0.36-1.56	0.69	0.31-1.53	1.65	0.84-3.24	1.71	0.85-3.44
Strongly to complete	0.55	0.30-1.01	0.76	0.39-1.48	0.60	0.35-1.05	0.72	0.40-1.30
**Sex**								
Male	1.00	-	1.00	-	1.00	-	1.00	-
Female	**2.94**	**1.80-4.81**	**3.08**	**1.80-5.24**	**1.95**	**1.32-2.88**	**1.83**	**1.20-2.79**
**Socioeconomic status**								
Lower	1.26	0.60-2.63	0.64	0.26-1.54	0.78	0.41-1.49	0.53	0.25-1.13
Middle	0.83	0.47-1.47	0.62	0.32-1.18	0.87	0.55-1.40	0.71	0.42-1.21
Upper	1.00	-	1.00	-	1.00	-	1.00	-
**Immigrant parent(s)**								
Both parents German	1.00	-	1.00	-	1.00	-	1.00	-
1 or 2 parents immigrant	**2.09**	**1.27-3.42**	1.64	0.91-2.97	1.23	0.80-1.90	1.12	0.66-3.34
**School type**								
Vocational	**2.70**	**1.39-5.26**	**2.25**	**1.00-5.09**	1.56	0.87-2.80	1.63	0.79-3.34
Intermediate	1.51	0.89-2.58	1.71	0.95-3.10	1.29	0.84-1.97	1.52	0.95-2.43
Academic	1.00	-	1.00	-	1.00	-	1.00	-

Note: Significant associations are emboldened.

**Table 7:** Association between suicidal ideation, attempted suicide and alternative identity

	**Suicidal ideation (n = 377)**				**Suicide attempt (n = 414)**			
**Predictors**	**OR**	**95% CI**	**Adj OR**	**95% CI**	**OR**	**95% CI**	**Adj OR**	**95% CI**
**Alt-identity**								
None	1.00	-	1.00	-	1.00	-	1.00	-
Moderate or stronger	**3.41**	**1.54-7.54**	**3.74**	**1.64-8.54**	**5.96**	**1.96-18.11**	**8.10**	**2.22-29.58**
**Jock-identity**								
None	1.00	-	1.00	-	1.00	-	1.00	-
Moderate	0.60	0.28-1.26	0.55	0.25-1.19	0.37	0.09-1.56	0.30	0.06-1.52
Strongly to complete	**0.49**	**0.27-0.90**	**0.51**	**0.27-0.98**	0.32	0.11-0.93	0.37	0.11-1.24
**Sex**								
Male	1.00	-	1.00	-	1.00	-	1.00	-
Female	**1.87**	**1.17-2.98**	**1.85**	**1.13-3.05**	1.81	0.69-4.77	2.00	0.67-5.91
**Socioeconomic status**								
Lower	1.00	-	1.00	-	1.00	-	1.00	-
Middle	0.92	0.43-1.94	0.93	0.39-2.22	1.42	0.19-10.35	0.64	0.07-5.79
Upper	0.70	0.41-1.20	0.70	0.39-1.26	2.50	0.56-11.24	2.00	0.37-10.78
**Immigrant parent(s)**								
Both parents German	1.00	-	1.00	-	1.00	-	1.00	-
1 or 2 parents immigrant	1.02	0.60-1.73	1.04	0.56-1.94	**4.65**	**1.76-12.32**	**6.22**	**2.01-18.67**
**School type**								
Vocational	0.75	0.33-1.69	0.63	0.24-1.66	4.29	0.70-26.33	2.01	0.27-37.30
Intermediate	0.99	0.61-1.60	0.99	0.58-1.69	**6.02**	**1.34-27.08**	**6.31**	**1.28-31.24**
Academic	1.00	-	1.00	-	1.00	-	1.00	-

Note: Significant associations are emboldened.

The association between Alternative identity and youth subculture was strengthened rather than attenuated by adjusting for covariates, e.g. (SHBQ unadjusted OR = 3.6 vs. adjusted OR = 4.2). We repeated the analysis using the three (Alternative, Indie and Urban) identity factor scores and found a similar pattern (Table 8). Even after adjustment for covariates, only the Alternative identity factor was consistently associated with self-harm, be that self-injury (SHBQ OR = 1.6; FASM OR = 1.5), suicidal ideation (OR = 1.3) or attempted suicide (OR = 2.5). Finally, adjusting for victimisation did not attenuate the association (results for categorical measured of identity shown in Table 9, results for factor measures of identity were similar and are available upon request).

**Table 8:** Association between self-harming behaviours, thoughts and subcultural identities

	**Self-injury (SHBQ, n = 387)**		**Self-injury (FASM, n = 376)**		**Suicidal ideation (n = 341)**		**Suicide attempt (n = 378)**	
**Identity factor**	**Unadj OR (95% CI)**	**Adj OR**^**1 **^**(95% CI)**	**Unadj OR (95% CI)**	**Adj OR**^**1 **^**(95% CI)**	**Unadj OR (95% CI)**	**Adj OR**^**1 **^**(95% CI)**	**Unadj OR (95% CI)**	**Adj OR**^**1 **^**(95% CI)**
Factor 1: Alt	**1.49 (1.17-1.90)**	**1.61 (1.23-2.12)**	**1.43 1.08-1.89)**	**1.51 (1.10-2.06)**	**1.29 (1.01-1.65)**	**1.31 (1.02-1.69)**	**1.89 (1.40-2.54)**	**2.48 (1.47-4.18)**
Factor 2: Indie	1.09 (0.86-1.38)	1.18 (0.91-1.52)	1.05 (0.85-1.30)	1.11 (0.88-1.40)	1.08 (0.85-1.37)	1.10 (0.86-1.40)	1.09 (0.74-1.74)	1.11 (0.62-2.01)
Factor 3: Urban	1.14 (0.90-1.44)	**1.37 (1.03-1.83)**	1.15 (0.94-1.42)	**1.43 (1.11-1.83)**	1.05 (0.82-1.35)	1.26 (0.95-1.67)	1.50 (0.92-2.28)	1.59 (0.84-3.00)

Note: Significant associations are emboldened.

^1^Adjusted for Jock identity, gender, socioeconomic status, immigrant background and school type.

**Table 9:** Association between self-harming behaviours, thoughts and Alternative identity adjusting for victimisation

	**SHBQ self-injury (n = 239)**		**FASM self-injury (n = 234)**		**Suicide ideation (n = 216)**		**Suicide attempt (n = 228)**	
**Identity**	**Adj OR**^**1 **^**(95% CI)**	**Victim OR**^**2**^**(95% CI)**	**Adj OR**^**1 **^**(95% CI)**	**Victim OR**^**2 **^**(95% CI)**	**Adj OR**^**1 **^**(95% CI)**	**Victim OR**^**2 **^**(95% CI)**	**Adj OR**^**1 **^**(95% CI)**	**Victim OR**^**2 **^**(95% CI)**
**Alt identity**								
No identification	1.00	1.00	1.00	1.00	1.00	1.00	1.00	1.00
Moderate or stronger	**2.63 (1.06-6.52)**	**2.90 (1.15-7.34)**	**3.12 (1.21-8.06)**	**3.20 (1.24-8.27)**	**3.16 (1.20-8.28)**	**3.01 (1.15-7.88)**	**6.70 (1.52-29.54)**	**7.40 (1.57-34.74)**

Note: Significant associations are emboldened.

^1^Adjusted for Jock identity, gender, socioeconomic status, immigrant background and school type.

^2^Adjusted for Jock identity, gender, socioeconomic status, immigrant background, school type and victimisation.

## Discussion

Our key finding is the confirmation of the ‘Alternative-identity’ effect, with around half of Alternative adolescents engaging in self-injury (NSSI) and around a 1 in 5 attempting suicide. We found a moderate sized correlation (r ≈ 0.3) between (Alternative) identity and self-harm, with Alternative teenagers between four to eight times more likely to engage in some form of self-harm than their peers. The other major goal of this study was to explore the potential reasons for these associations.

The Alternative-identity effect fulfils many of the standard epidemiological assessment criteria regarding causality [47] such as consistency, dose–response relationship, strength of association, specificity to mental health and the fact that it is unaccounted for by artefacts or confounders. However other key causal criteria such as plausibility, temporal relationship and alternative explanations remain understudied. Against our expectation, adjusting for confounders strengthened the Alternative-identity effect. While we may have omitted important confounders, adjusting for a range of risk-factors such as socioeconomic status, area, substance use, etc. only strengthens the effect [9].

### Youth identity and self-harm

In contrast to the Alternative-identity effect, a ‘Jock’ identity is somewhat protective against self-harm. This may be attributable to a combination of their high peer-status [27,29,48] and regular physical exercise [9], both linked to improved psychological health [49]. This finding is in line with research showing that a positive coping style is negatively associated with NSSI [50]. Surprisingly, and counter to our predictions, identifying as a Nerd is unrelated to indicators of psychological health such as NSSI, suicidal ideation or attempted suicide. This indicates a shift upwards from their previously marginalised status and demonstrates it is possible to improve the reputation of even a heavily stigmatised adolescent identity within a generation [51,52].

The association between different forms of self-harm and Alternative identity appears robust irrespective of its nature. However, there are qualitative differences between suicidal and NSSI, with the later the main focus of our study. Accordingly, one of our key aims is to understand ‘why’ Alternative teens engage in NSSI. Not only do Alternative youth self-injure more often than non-Alternative teens, their underlying motivations are different to other self-injurers. Alternative teenagers engage in more frequent acts of self-injury, have stronger motivation to self-injure (i.e. score higher on all but one of the FASM motivation for self-harm items) and frequently endorse both automatic and social reasons for self-injury. While Alternative teenagers are more likely to use self-injury to communicate distress or seek help they frequently self-injure to regulate their emotions. Thus, the stereotype of attention seeking Goth or Emo teenagers is only partially supported. Alternative teenagers are more likely to use self-injury to ‘belong to a group’ and ‘to avoid being with people’. Although speculative this pattern implies a strong in/out-group outlook is prevalent within Alternative youth culture with self-injury perceived by a minority of Alternative teenagers as a defining group characteristic. This is compatible with a social or self-identity interpretation of the phenomena.

### Youth identity and peer contagion of self-harm

Having established that the Alternative-identity connection with self-harm is robust and eliminated the idea that this is merely an artefact attributable to social background. How do we integrate this work into current theoretical models of self-harm and social contagion? Our study is in part exploratory and thus is not conclusive in its findings. That said, we can speculate about the potential mechanisms that may explain the strong Alternative-identity effect we report. Without longitudinal data it is difficult to justify favouring any one social contagion mechanism [34], but in general our results are more consistent with the *assortive relations* mechanism. The repetitive nature, severity of self-harm and its key role in regulating emotions is probably underpinned by neurobiological individual differences [23,53] rather than peer influence. The stereotypical ‘Alternative teen’ temperament and personality profile, i.e. an introverted, neurotic, impulsive, risk-taker with nonconformist tendencies [7] features several known personality risk factors for NSSI [54]. For example, openness to experience (nonconformity) predisposes individuals to experiment with different coping strategies, including self-injury [55]. High levels of neuroticism are also linked to NSSI [56,57]. Feelings of loneliness, isolation and alienation from peers and wider society are all major components of contemporary suicide and self-injury theories [58-60]. It is premature to dismiss the role of *direct* or *indirect imitation* given that a minority of Alternative teenagers report they self-injure in order to ‘belong to a group’. The two key question for researchers investigating social modelling [34] are firstly to determine if such influence is primarily direct (peer-group) or indirect (media), and secondly to establish what proportion of ‘social self-injurers’ progress to engaging in more severe or chronic self-injury; if the proportion is high then further study is a priority.

The clustering of self-harm among Alternative youth may paradoxically indicate that membership serves several positive psychological functions and ‘belongingness’ to a certain youth group could be viewed as protective [61]. Firstly, given isolation, alienation, social disconnection and ‘thwarted belongingness’ are major predictors of self-harm, identifying with a group who share similar interests and values should reduce alienation and may help buffer the effects of low peer status and stigmatisation [29]. Secondly, from a developmental perspective, establishing a personal identity and a set of core personal values - as distinct from one’s parents - is a critical life-stage and belonging to the (Alternative) peer group may perform a key role in developing a sense of independence, mastery and control [62]. Thirdly, given the personal characteristics ascribed to Alternative teenagers such as intelligence, nonconformity and creativity, but also introversion and a melancholic and morbid outlook [7] their peer group provides an environment in which such characteristics can be expressed without stigma. For example in most schools scholastic ability is associated with low peer-status [48], while within the Alternative subculture it (alongside intelligence) is valued [7,28,63]. Similarly low self-esteem is a putative NSSI vulnerability factor [64] and group membership arguably boosts self-esteem [65]. This disconnect from wider peer norms may explain the strong social role that NSSI serves within the Alternative peer group, both to separate it from other groups, e.g. ‘avoid being with people’ and as characteristic in-group feature for some, e.g. ‘to belong to a group’. Finally, the strong communicative function of NSSI suggests self-injury is regarded as a valid method of soliciting help from other members of the group during periods of psychological distress. This may have mixed consequences, both facilitating help-seeking behaviour and unintentionally reinforcing self-injurious behaviour.

### Limitations, recommendation for future research and clinical implications

This study has a number of methodological limitations. The cross-sectional nature of the study limits our ability to make causal inference. The moderate sample size, relatively small number of Alternative teenagers and the brief nature of assessment suggest the study has modest statistical power. The lack of available information on missing data restricts our ability to generalise our findings to pupils unable or unwilling to participate. Our reliance on self-report means we may have somewhat reduced reliability regarding self-harming behaviours (see below) or social confounders, but is irrelevant for self-identity measures. Our measure of subcultural identity needs further validation. This is inherently problematic given the shifting nature of youth culture and while some youth cultures are relative stable any measure of subcultural identity will require adaptation to local context.

Surprisingly, the FASM 1-year NSSI prevalence (45.7%) is higher than the lifetime NSSI rate provided by the SHBQ (20.7%), yet this counterintuitive finding is attributable to its longer length, the more comprehensive list of self-injury methods included in the FASM and is consistent with previous findings. While we have explored how Alternative youth differ in their motivations for NSSI, we have not explored their motivations for suicide. It remains to be established which of the two common methods of measuring subcultural identity, factor scores or categorical measures, is superior. Importantly, our findings are robust irrespective of the method used. Despite these limitations the ability to link subcultural affiliation with a number of features of self-harm, particularly motivations, is novel and provides further insight into the nature of the relationship.

Future work should explore if this is exclusively a western phenomena, although the existence of similar ‘dark’ and morbid but non-western subcultures such as Gothic Lolita [66] (membership of which has been anecdotally linked with psychological trauma [67]) suggests not. Complimentary qualitative and mixed methods studies could provide invaluable ‘in depth’ insights into the social and personal motivations of Alternative teenagers suicidal and self-injuring behaviours than psychometric measures alone. We extended this research topic by incorporating the peer crowd (e.g. Jocks and Nerds) framework and this requires replication. In the light of our reversal of ‘Nerd’ subculture from marginalised to revered status finding, a time series analysis of the Alternative-identity effect is warranted to determine if the effect is stronger among particular generations.

Another key question is related to intervention; given that the Alternative-identity effect is one of the largest effects thus found in NSSI research, is it possible to design peer-group appropriate interventions? One intervention involves knowledge exchange and training to assist educators in understanding the nature and function of different youth subcultures. At least two organisation use this form of intervention; the UCLA Center for Mental Health in Schools [68] and the Sophie Lancaster Foundation [69], with the former aiming to improving mental health and the latter aiming to reduce victimisation. Peer-based interventions for smoking prevention use social learning mechanisms; by training popular ‘peer-leaders’ to disseminate anti-smoking information they capitalise on modelling behaviour and peer status [70]. Given the strong link between self-harm and peer affiliation [71] this intervention model may be particularly effective in relation to self-injury, but could unintentionally aggravate NSSI social contagion. Clinicians may consider treatments which complement adolescents’ existing peer identities: we speculate that such ‘matching’ may improve treatment effectiveness. For example, although the effectiveness of music therapy in treating NSSI is unproven, Alternative teenagers are particularly receptive to this form of treatment [43] and it follows that ‘Nerds’ may be more receptive to online [72] and Jocks to exercise-based intervention [73]. Others see identifying adolescents particularly vulnerable to social contagion as a priority [34] and identifying the minority of teenagers who use self-injury as a marker of identity is a related priority.

## Conclusions

This study provides new evidence of the link between Alternative identity and self-harm. It suggests NSSI’s communicative role among this group and its minor, but significant, role in forming group identity. It is surprising that such a robust and strong effect is so understudied within adolescent psychiatry and clinical psychology. Sociology and social, developmental, health and consumer psychology recognise the importance of subcultural identity. Our work illustrates how much identity contributes to adolescents’ self-harming behaviour and the extent to which research on this issue has the potential to inform future interventions.

## Competing interests

PLP is involved in a clinical study sponsored by Lundbeck pharmaceuticals. All authors declare that they have no competing interests.

## Authors’ contributions

RY conducted the literature search, data analysis, drafted the article and designed the sociometric-subcultural component of the study. PLP and NS designed the main study, its methodology and collected the primary data. RCG and MP contributed to the study, collected data, developed the socioeconomic status variable and conducted translations. All of the authors read and approved the final manuscript, contributed to the literature search and interpretation of data.

## Pre-publication history

The pre-publication history for this paper can be accessed here:

http://www.biomedcentral.com/1471-244X/14/137/prepub

## Supplementary Material

Additional file 1: Table S2 Correlation between self-injury, suicidal thoughts or behaviours. Click here for file

Additional file 2: Table S1 Reason for self-injury by Alternative (Emo, Punk, Goth) identification. Click here for file
