# Supplementary material for: Why alternative teenagers self-harm: exploring the link between non-suicidal self-injury, attempted suicide and adolescent identity
Source: BMC Psychiatry. 2014 May 22;14:137. doi: 10.1186/1471-244X-14-137 (PMC4067739; doi:10.1186/1471-244X-14-137)
Supplement: Additional file 1: Table S2 — Correlation between self-injury, suicidal thoughts or behaviours. [file 1471-244X-14-137-S1.docx]

## Additional file 1: Table S2 - Correlation between self-injury, suicidal thoughts or behaviours.

| **No.** | **Identity or self-harm measures** | **1.** | **2.** | **3.** | **4.** | **5.** | **6.** |
| --- | --- | --- | --- | --- | --- | --- | --- |
| 1. | SHBQ Self-injury | - |  |  |  |  |  |
| 2. | SHBQ Self-injury frequency | .81^**^ | - |  |  |  |  |
| 3. | FASM Self-injury | .49^**^ | .45^**^ | - |  |  |  |
| 4. | FASM Self-injury frequency | .52^**^ | .60^**^ | .75^**^ | - |  |  |
| 5. | SHBQ Suicide attempt | .33^**^ | .38^**^ | .17^**^ | .23^**^ | - |  |
| 6. | SHBQ Suicide ideation | .29^**^ | .40^**^ | .27^**^ | .27^**^ | .17^**^ | - |

Note: leading zeros omitted. * = p-level ≤ .05, ** = p-level ≤ .01. N = 352.
