# Supplementary material for: Why alternative teenagers self-harm: exploring the link between non-suicidal self-injury, attempted suicide and adolescent identity
Source: BMC Psychiatry. 2014 May 22;14:137. doi: 10.1186/1471-244X-14-137 (PMC4067739; doi:10.1186/1471-244X-14-137)
Supplement: Additional file 2: Table S1 — Reason for self-injury by Alternative (Emo, Punk, Goth) identification. [file 1471-244X-14-137-S2.docx]

## Additional file 2: Table S1 - Reason for self-injury by Alternative (Emo, Punk, Goth) identification.

|  | **Non-alt identity (n=145)** | |  | **Alt identity (n=22)** | | **Mann-Whitney U (exact 2-tail)** | | |
| --- | --- | --- | --- | --- | --- | --- | --- | --- |
| **Reasons for self-injury** | **Mean ranks** | **Sum of ranks** |  | **Mean ranks** | **Sum of ranks** | **Z** | **p-level** | **Corrected**  **p-level** ^1^ |
| 8. To receive more attention from your parents or friends | 85.59 | 13095.5 |  | 104.75 | 2304.5 | -3.36 | **.006** | **.021** |
| 17. To get your parents to understand or notice you | 86.94 | 13215.5 |  | 91.34 | 2009.5 | -1.49 | .518 | .681 |
| 11. To get other people to act differently or change | 87.13 | 13331.5 |  | 94.02 | 2068.5 | -2.53 | .229 | .357 |
| 15. To let others know how desperate you were | 84.15 | 12706.5 |  | 106.57 | 2344.5 | -3.72 | **.006** | **.021** |
| 20. To get help | 85.97 | 13153.0 |  | 102.14 | 2247.0 | -4.28 | **.002** | **.019** |
| 12. To be like someone you respect | 87.93 | 13453.5 |  | 88.48 | 1946.5 | -0.26 | 1.000 | 1.000 |
| 3. To get attention | 85.52 | 13085.0 |  | 105.23 | 2315.0 | -3.81 | **.004** | **.021** |
| 21. To make others angry | 86.87 | 13291.0 |  | 95.86 | 2109.0 | -1.33 | .054 | .097 |
| 14. To stop bad feelings | 85.74 | 13204.5 |  | 107.80 | 2371.5 | -2.22 | **.028** | .058 |
| 7. To try to get a reaction from someone, even if it’s negative | 85.75 | 13034.0 |  | 99.59 | 2191.0 | -2.01 | **.023** | .051 |
| 22. To feel relaxed | 86.84 | 13286.5 |  | 96.07 | 2113.5 | -1.12 | .164 | .273 |
| 6. To get control of a situation | 86.88 | 13292.5 |  | 95.80 | 2107.5 | -1.18 | .304 | .447 |
| 4. To feel something | 84.02 | 12854.5 |  | 115.70 | 2545.5 | -4.07 | **≤.001** | **.004** |
| 10. To punish yourself | 86.07 | 13255.0 |  | 105.50 | 2321.0 | -2.81 | **.018** | **.049** |
| 2. To relieve feeling numb or empty | 85.49 | 13166.0 |  | 109.55 | 2410.0 | -3.38 | **.001** | **.013** |
| 18. To give yourself something to do when alone | 86.82 | 13197.0 |  | 92.18 | 2028.0 | -0.44 | .477 | .663 |
| 13. To avoid punishment or paying the consequences | 87.86 | 13443.0 |  | 88.95 | 1957.0 | -0.46 | 1.000 | 1.000 |
| 9. To avoid being with people | 86.40 | 13219.5 |  | 99.11 | 2180.5 | -3.13 | **.008** | **.026** |
| 5. To avoid doing something unpleasant you don’t want to do | 86.20 | 13189.0 |  | 100.50 | 2211.0 | -1.88 | **.031** | .060 |
| 16. To feel more a part of a group | 86.22 | 13191.5 |  | 100.39 | 2208.5 | -3.27 | **.005** | **.021** |
| 1. To avoid school, work, or other activities | 85.82 | 13045.0 |  | 99.09 | 2180.0 | -2.31 | **.020** | **.049** |
| 19. To give yourself something to do with others | 87.93 | 13366.0 |  | 84.50 | 1859.0 | 0.88 | .606 | .758 |

Items scored on a 4-point 0-3 scale. Significant differences are emboldened.

^1^ Benjamini-Hochberg corrected p-values for multiple testing.

## 
